# Comparison of HercepTest™ mAb pharmDx (Dako Omnis, GE001) with Ventana PATHWAY anti-HER-2/neu (4B5) in breast cancer: correlation with *HER2* amplification and HER2 low status

**DOI:** 10.1007/s00428-022-03378-5

**Published:** 2022-08-16

**Authors:** Josef Rüschoff, Michael Friedrich, Iris Nagelmeier, Matthias Kirchner, Lena M. Andresen, Karin Salomon, Bryce Portier, Simone T. Sredni, Hans Ulrich Schildhaus, Bharat Jasani, Marius Grzelinski, Giuseppe Viale

**Affiliations:** 1 Targos - A Discovery Life Sciences Company, Germaniastraße 7, 34119 Kassel, Germany; 2 Institute of Pathology Nordhessen, Germaniastraße 7, 34119 Kassel, Germany; 3 Agilent Technologies Denmark Aps, Produktionsvej 42, 2600 Glostrup, Denmark; 4 Agilent Technologies Inc, 5301 Stevens Creek Blvd, Santa Clara, CA 95051 USA; 5 IEO European Institute of Oncology IRCCS, and University of Milan, Milan, Italy

**Keywords:** Invasive breast carcinoma, HER2, IHC, FISH, HercepTest (mAb), Dako Omnis, PATHWAY 4B

## Abstract

Performance of the new CE-IVD-marked HercepTest™ mAb pharmDx (Dako Omnis) assay (HercepTest (mAb)) was compared against the PATHWAY® anti-HER-2/neu (4B5) (PATHWAY 4B5) assay using 119 pre-selected breast cancer samples covering the entire range of HER2 immunohistochemistry (IHC) expression scores (0, 1 + , 2 + , 3 +). The sensitivity and specificity of both assays were assessed based on consensus IHC scores and amplification status, as determined by fluorescence in situ hybridization (FISH) according to 2018 ASCO/CAP testing guidelines. There was a high concordance between results from the HercepTest (mAb) and PATHWAY 4B5 assays for HER2-negative (IHC 0, 1 + , 2 + and FISH negative) and HER2-positive (IHC 3 + , 2 + and FISH positive) breast carcinomas (98.2%). Regarding individual IHC scores, complete agreement was achieved in 69.7% (83/119) of cases, and all but one of the discordant cases were due to higher HER2-status scoring using the HercepTest (mAb). Thus, more tumors were overscored as IHC 2 + by HercepTest (mAb) (27 *versus* 15) as evidenced by their lower FISH positivity rate (48.1% *versus* 80%). However, two amplified tumors identified as IHC 2 + by HercepTest (mAb) were missed by PATHWAY 4B5 (IHC 1 +). Four additional cases identified as IHC 2 + by HercepTest (mAb), with FISH ratio < 2 but elevated gene counts (≥ 4 to < 6), were recorded negative by PATHWAY 4B5. The HercepTest (mAb) detects HER2 expression with higher sensitivity in tumors with gene amplification (ISH group 1) and increased gene counts (ISH group 4) as well as in HER2-low tumors (HER2 IHC2 + /FISH negative or IHC 1 +). Future studies will demonstrate whether this translates into improved patient selection especially for new HER2-directed therapies.

**Supplementary Information:**

The online version contains supplementary material available at 10.1007/s00428-022-03378-5.

## Introduction

The human epidermal growth factor receptor 2 (HER2, also referred to as HER2/neu) is one of four members belonging to the epidermal growth factor receptor (EGFR) protein family. The HER2 protein is characterized by its tyrosine kinase activity and the *HER2* oncogene controls cell proliferation and apoptosis [1].

Initially described in 1985 by King et al. [2], HER2 overexpression has been demonstrated to play a major role in the onset, development, and progression of breast cancer (BC). About 15–20% of BC patients show *HER2* amplification and/or HER2 over-expression, which are associated with increased tumor aggression and poor prognosis, although these patients are eligible for HER2-directed therapy [3–6]. HER2-targeted monoclonal antibodies (mAbs) such as trastuzumab and/or pertuzumab (used as single or combined agents, with or without chemotherapy) are now the standard treatment for patients with HER2-positive advanced BC, acting to block the corresponding pathway(s) and provide improved overall survival rates [7]. Beyond the use of these two drugs, novel therapies based on anti-HER2 antibody–drug conjugates (ADCs) have been developed. For example, trastuzumab-emtansin (T-DM1, Kadcyla®) was the first of its kind approved in 2013 by European Medicines Agency (EMA) for HER2 overexpressing and/or amplified advanced metastasized BC [8]. New types of ADCs have recently been developed using trastuzumab linked to novel toxic agents (e.g., deruxtecan, a topoisomerase I inhibitor) (T-DXd, Enhertu®) and have shown efficacy in patients even after T-DM1 therapy failure [9]. Interestingly, there is also evidence that T-DXd is effective in patients with BC exhibiting low levels of HER2 protein as determined by IHC (i.e., HER2 IHC 2 + /non-amplified or IHC 1 +) [10]. Since, almost 40–50% of BC are classifiable as HER2-low [3, 4], many more patients may benefit from this new type of HER2-targeted therapy (reviewed in [11]).

Methods to screen for eligible BC patients who may benefit from HER2-targeted therapies currently include IHC demonstrating HER2 protein overexpression and in situ hybridization (ISH) to detect *HER2* gene amplification. Other methods such as quantitative real-time PCR (transcript amplification) are not recommended for routine patient selection [12]. Available IHC assays are well-established, robust, and inexpensive. While several different antibody clones have been successfully used in clinical trials (e.g., R60, 10H8, and CB11), the Agilent/Dako HercepTest™ pAb pharmDx (Autostainer Link) (HercepTest (poly)) and Ventana PATHWAY® anti-HER-2/neu (4B5) (PATHWAY 4B5) are currently the most widely used IHC assays [13, 14]. Many studies have analyzed the diagnostic value (i.e., sensitivity and specificity) of these two IHC assays for detecting HER2-positive BC by comparing IHC results to the *HER2* gene amplification status determined by ISH assays [15–17]. Accordingly, international guidelines for HER2 testing in BC [18] focus on the correlation between IHC and ISH to reliably select those HER2-positive carcinomas most likely to respond to HER2-directed therapies.

Due to the potential broader applicability of current anti-HER2-targeting drugs, the sensitivity of these assays is now of greater importance for selecting eligible patients [11]. In this context, it has become necessary to evaluate the diagnostic utility of HER2 assays with respect to the detection of not only HER2-positive (IHC 3 + and/or amplified) but also HER2-low (IHC 2 + or IHC 1 + , non-amplified) BC cases. In this context, studies comparing the polyclonal HercepTest (poly) and the monoclonal PATHWAY 4B5 have revealed good concordance between the two methods for detection of HER2-positive BC [16, 17]. However, there is evidence that the HercepTest (poly) might be less sensitive in detecting HER2-low BC as compared to the PATHWAY 4B5 assay [19]. Recently, a second-generation, CE-IVD-marked HercepTest™ mAb pharmDx (Dako Omnis) kit (HercepTest (mAb)) has become available in Europe and Canada. This new assay is run on the Dako Omnis staining platform using a monoclonal rabbit antibody (clone DG44) [20]. Interestingly, according to the 2021 NordiQC data, laboratories applying the HercepTest (mAb) achieved highest overall pass rate (100%) [21].

Herein, we report the results of an IHC concordance study comparing the HercepTest (mAb) run on the Dako Omnis platform and the PATHWAY 4B5 assay run on the Ventana BenchMark ULTRA using a BC cohort of 119 samples and assessing assay sensitivity and specificity with respect to amplification status and inter-assay and inter-observer variations.

## Materials and methods

### *Sample selection and study design (Fig. **1**)*

**Fig. 1:**
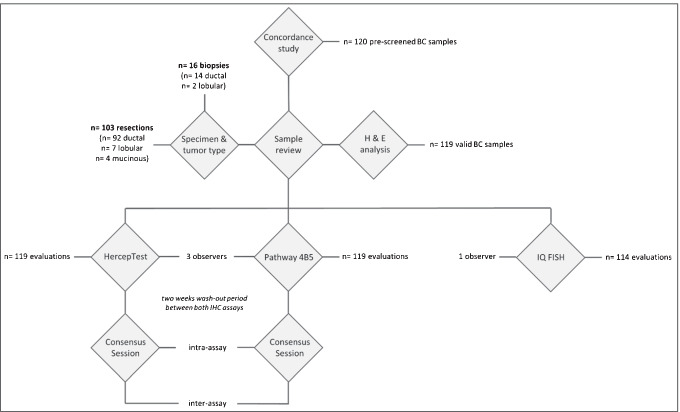
Schematic illustration of the study design

The clinical performance of the new HercepTest (mAb) (Agilent Technologies, Santa Clara, CA. USA) was compared with the monoclonal PATHWAY 4B5 assay performance (Ventana) (Roche Tissue Diagnostics, Tucson, AZ. USA) using a selection of 120 BC samples. These commercially acquired tissue blocks were originally pre-tested for their HER2 status by the vendor using either Ventana or Leica antibodies and verified by Agilent Technologies applying HercepTest (mAb) and HercepTest (poly). The testing cohort composed of an equal number of cases representative of HER2 status 0, 1 + , 2 + , and 3 + , respectively (*n* = 30/status). Within the IHC score 2 + group, 15 samples were selected to be *HER2* amplified, while the remaining samples were non-amplified. Specimens were acquired by Agilent Technologies from Danish hospitals (with ethical permission) and external tissue vendors in USA and Canada (see Vendor list). The specimens were de-identified, and all were fixed in 4% neutral buffered formalin and paraffin-embedded compliant with ASCO/CAP guidelines.

The BC specimens were enrolled in the study following assessment of tissue sections stained with H&E, HercepTest (mAb), and HercepTest (poly). A specimen was included if (1) invasive BC tissue and an adequate number of tumor cells (≥ 100) were present, (2) the tissue morphology was adequately preserved, and (3) there was an absence of processing artifacts that would negatively affect the assessment of the HER2 status. Each specimen entered the study with an enrollment IHC score based on HercepTest (mAb) and HercepTest (poly). FISH status for enrollment of amplified and non-amplified specimens was based on information previously provided by the commercial tissue vendor, if these data were available.

One tumor sample had an inadequate amount of tumor tissue and was rejected by the observing pathologists; hence, a total of 119 BC specimens were used for this study. The final selection of samples consisted of 103 surgical resections and 16 biopsy specimens. Tumor types included 106 ductal (89.1%), 9 lobular (7.6%), and 4 mucinous (3.4%) carcinomas. IHC results were assigned to each of these 119 samples. *HER2* FISH analysis revealed 114 evaluable samples out of 119 tested (see also Supplementary Data 1); five of the BC samples produced non-evaluable FISH signals due to sub-optimal tissue pre-analytics despite repeat testing.

### Sample preparation

Twelve tissue sections, 4–5 µm thick, cut from each of the selected specimens were mounted onto Epredia™ SuperFrost Plus™ Microscope Slides. On-slide controls containing HER2-positive (FFPE cell pellet from IHC 2 + cell line MDA-453) and negative (tonsil sample) cores were added to each slide. Mounted tissues were baked at 60 °C for 1 h. Two tissue sections (first and last) from each collected specimen were H&E stained.

### Immunohistochemistry

#### HercepTest™ mAb pharmDx (Dako Omnis) (GE001)

The IHC staining protocol using the HercepTest (mAb) was performed as described by the manufacturer [20]. Freshly cut tissue was processed on the Dako Omnis platform (Agilent Technologies, Santa Clara, CA) together with kit control slides for every staining run, using an automated staining protocol validated for HER2 detection [20].

#### PATHWAY® anti-HER-2/NEU, clone 4B5 (790–2991)

IHC staining using the PATHWAY® HER-2/neu rabbit monoclonal antibody 4B5 was performed according to the recommendations of the manufacturer [22]. Freshly cut tissue was processed on the Ventana BenchMark ULTRA (Ventana Medical Systems, Roche Diagnostics, Tucson, AZ) together with kit control slides for every staining run, using an automated staining protocol validated for HER2 detection [22].

#### IHC scoring

IHC staining for HER2 was independently evaluated by three trained pathologists (IN, MK, JR), followed by a consensus session for discordantly scored samples to define a consensus score for each case. IHC stains of the two assays were read after a 2-week wash-out period, and all the pathologists were blinded to the FISH results. In addition to a pre-study training provided by Dako/Agilent, all investigators had extensive experience in HER2 evaluation, having served over the past 20 years as readers in most of the trastuzumab, pertuzumab, and T-DM1 approval BC studies by Targos GmbH (Kassel, Germany) (for a review of studies screened by first-generation HercepTest (poly), see [23]).

IHC scoring was performed according to the 2018 ASCO/CAP guidelines [18]. Accordingly, cases with complete intense staining in ≤ 10% of tumor cells, as well as cases with intense and lateral or basolateral (“U-type”) staining, were included in the IHC 2 + category. For cases of IHC 1 + staining intensity (i.e., faint/barely perceptible membrane staining), the percentage of stained cells ≤ 10% or > 10% was recorded separately according to Ventana Instructions for Use (IFU) [22]. Intensity scoring was performed by applying the magnification rule as published previously by our group [24, 25].

### *Fluorescence *in situ* hybridization assessment*

#### HER2 IQFISH pharmDx (K5731)

Determination of *HER2* gene amplification was analyzed using the *HER2* IQFISH pharmDx kit according to the recommendations of the manufacturer [26]. *HER2* in situ hybridizations were evaluated by a pathologist (IN) using the updated 2018 ASCO/CAP guidelines. For final interpretation of the FISH data, newly defined ISH groups (1–5) were taken into consideration [18, 27]. Accordingly, group 1 (ratio ≥ 2.0 and gene count ≥ 4.0) and group 3 cases (ratio < 2.0 and gene count ≥ 6.0) with IHC 3 + or IHC 2 + were considered FISH positive.

### Statistical evaluations

For comparison of datasets, chi-square test (*X*^2^) was used with *p* < 0.05 considered as statistically significant.

Test performance was evaluated using FISH as a reference standard. Sensitivity and specificity were calculated as follows:

$$\mathrm{Estimated sensitivity}=100\mathrm{\%}\times \frac{\#\mathrm{ true positive events}}{\#\mathrm{ true positive events}+\#\mathrm{ false negative events}}$$

$$\mathrm{Estimated specificity}=100\mathrm{\%}\times \frac{\#\mathrm{ true negative events}}{\#\mathrm{ false positive events}+\#\mathrm{true negative events}}$$

Inter-rater reliability (IRR), defined as the ratio of the total number of agreements among raters and the total number of ratings, was calculated as follows:

$$\mathrm{IRR }\left[\mathrm{\%}\right]=\frac{\mathrm{Total }\#\mathrm{ of agreements}}{\mathrm{Total }\#\mathrm{ of ratings given by each rater}*\#\mathrm{ of raters}}\times 100$$

## Results

### *Performance of HercepTest (mAb) and inter-rater agreement (Fig. **2**)*

**Fig. 2:**
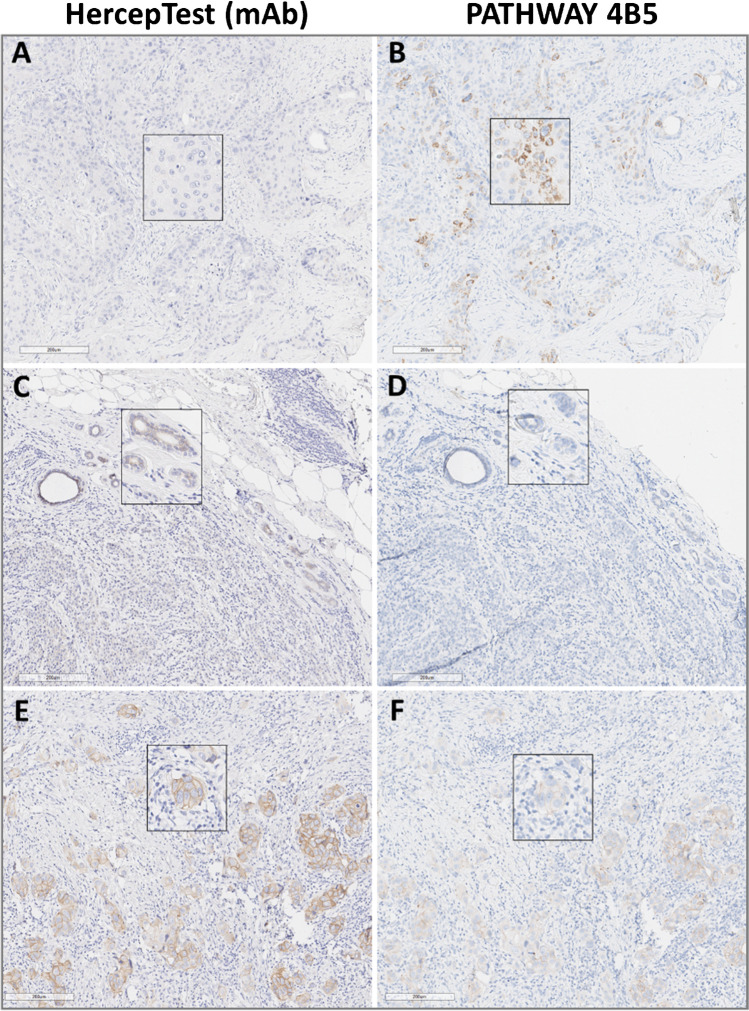
Comparison of HER2 detection by both IHC assays (HercepTest (mAb) versus PATHWAY 4B5). A, B Non-specific cytoplasmic granular staining by using PATHWAY 4B5 not visible by using HercepTest (mAb). C, D Weak to moderate staining within some accompanying normal duct epithelium by using HercepTest (mAb), barely visible by using PATHWAY 4B5. E, F Comparison of discordantly scored (HercepTest (mAb) IHC 2 + , PATHWAY 4B5 IHC 1 +) in a FISH HER2-positive sample (#86). Scale bar: 200 µm (magnification 10 ×). Inserts show enlargements of the respective photomicrographs (magnification 20 ×)

In HER2-expressing samples, each of the HER2 IHC assays produced specific membrane-bound staining that was easy to interpret at all intensities (weak to strong). Although non-specific background staining was not observed, a weak and only focally distributed staining of normal duct cells was detected with HercepTest (mAb) (Fig. 2A, C, ). Furthermore, PATHWAY 4B5 staining was characterized by the occasional presence of diffuse and/or dot-like cytoplasmic staining in tumor and normal cells, as previously reported [28]. Signal detection in normal duct samples was usually of low intensity (Fig. 2B, D, ). Noteworthy, we did not observe relevant staining differences between the sample types, e.g., no higher frequency of edge artifacts in biopsies.

Within the HercepTest (mAb) and the PATHWAY 4B5 assays, an overall inter-reader agreement of 84% (100/119) and of 89.1% (106/119) was observed. Study IRR was recorded as 89.4% and 92.7%, respectively. Discrepantly scored samples were re-evaluated by all three observers during a final review session and assigned consensus scores that were used for further analyses.

It is noteworthy that most disagreements (68.8%) between pathologists’ scores were observed within the HER2-low range (later consented as IHC score 0 or 1 +), especially near the cut-off for HER2 ultra-low category exhibiting a HER2 score of 0 with incomplete and faint staining in ≤ 10% of tumor cells. This led to several challenging samples around the cut-off value (IHC 1 + versus IHC 0, according to ASCO/CAP 2018).

### *HercepTest (mAb) and PATHWAY 4B5 — inter-assay concordance (*Table 1*)*

**Table 1:**
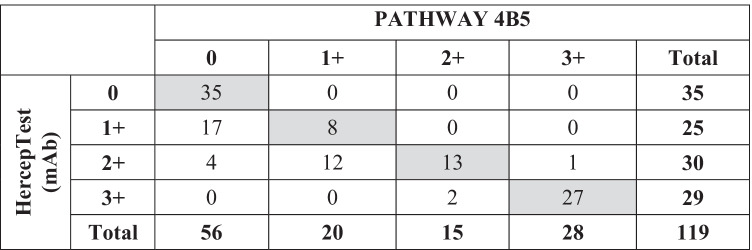
Comparison of HER2 scorings derived from the indicated IHC assays

Based on the consented scores for both assays, complete concordance was reached in 83 of 119 tumors (69.7%). Thirty-six samples received discordant scores, including 26 resections (25.2%) and 10 biopsies (62.5%). Virtually, all these cases (*n* = 35) showed higher scores with HercepTest (mAb), and in only one case (biopsy) was the staining recorded to be higher by PATHWAY 4B5. While 56 samples were evaluated as negative (IHC 0) for HER2 by PATHWAY 4B5, only 35 specimens were likewise identified by HercepTest (mAb). Thus, adjustments to discordant scores were mainly associated with the PATHWAY 4B5 negative group of IHC 0 and IHC 1 + (33 of 36). This led to a significantly different classification of BC samples by both assays. For instance, the group of HER2-low expressing samples (HER2 score 2 + or 1 + /not amplified) was found to be significantly larger for HercepTest (mAb) (35% versus 19%; *p* < 0.01).

Only three of the discordant cases were observed in the PATHWAY 4B5 IHC 2 + and IHC 3 + group, with scores for two tumors being raised from IHC 2 + to 3 + , and one score downgraded from IHC 3 + to 2 + . Lastly, the concordance of both assays was found to be 83.7% (87/104 cases) for HER2-negative (IHC 0/1 +) versus HER2-positive (IHC 3 +).

### *HercepTest (mAb) and PATHWAY 4B5 — correlation with FISH (Fig. **3A, B**)*

**Fig. 3:**
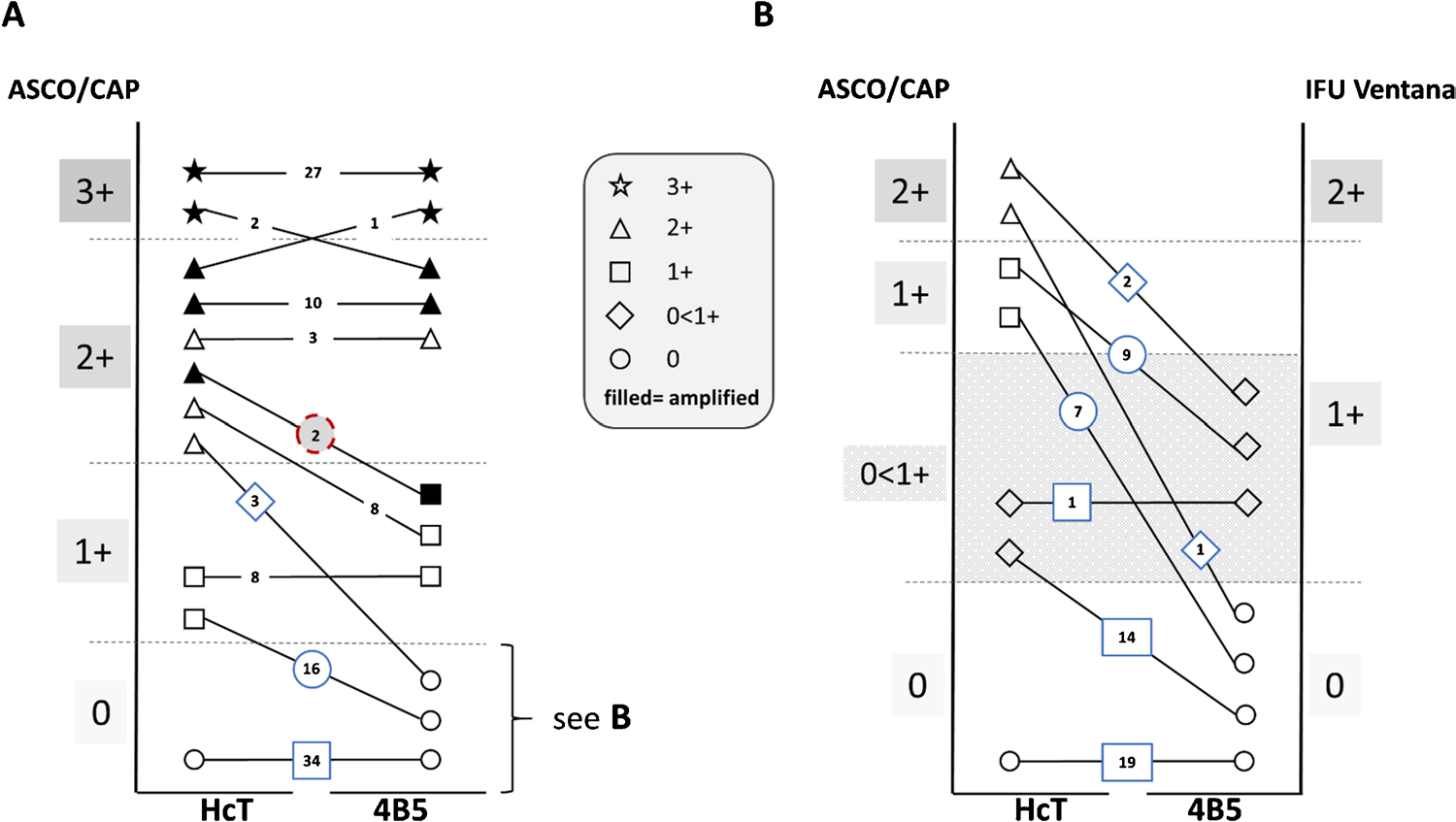
Comparison of HercepTest (mAb) (HcT) and PATHWAY 4B5 (4B5) with respect to IHC scores and FISH status. A According to ASCO/CAP IHC scoring, all ISH positive cases (filled symbols) were scored IHC 3 + or IHC 2 + by using HcT. In two of the ISH positive tumors, 4B5 was IHC 1 + (red circle). B Detailed analysis of 53 tumors scored as IHC 0 according to ASCO/CAP by 4B5 (see cases within blue frames in A) with 41 tumors showing no staining and 12 cases with HER2 expression in < 10% of cells (grey area). Using HcT, 16 cases were shifted up to IHC score 1 + and in 3 cases to IHC 2 + (matching to HER2-low category). Fourteen cases were shifted from no staining by 4B5 to some staining (< 10%, grey zone) by HcT (matching to HER2 ultra-low category: 0 < 1 +). IHC scores were unchanged in 20 cases (19 IHC 0, 1 IHC 0 < 1 +)

FISH data were obtained for 114 specimens, 42 of which showed *HER2* amplification (Fig. 3A). All non-amplified cases (*n* = 72) were identified as IHC negative (0/1 +) or equivocal (2 +) by both assays, i.e., no false positives were recorded, corresponding to 100% specificity. However, two false negatives were observed with the PATHWAY 4B5 assay in which two amplified (surgical) specimens showed an IHC 1 + score compared to a IHC 2 + score with the HercepTest (mAb), leading to a slightly lower sensitivity for PATHWAY 4B5 (95.2% versus 100%; Fig. 2E, F). Both cases were tested amplified according to the external vendor information as well as within this study. Although more IHC 2 + cases were identified by the HercepTest (mAb) as being not amplified (14 of 27) compared to PATHWAY 4B5 (3 of 15), all the amplified tumors were detected as positive (IHC 2 + or 3 +) when using the HercepTest (mAb) (see Figs. 2E, F and 3A).

A more detailed analysis of FISH data was conducted with respect to ISH groups according to ASCO/CAP 2018 guidance [18]. Compared to PATHWAY 4B5, scores for 13 cases were increased to IHC 2 + when using HercepTest (mAb) (*n* = 10 from IHC 1 + and *n* = 3 from IHC 0; see Table 2). In two tumors, FISH revealed a *HER2* ratio ≥ 2.0 and mean gene count per cell ≥ 4.0, corresponding to ISH group 1 (HER2 positive; see Fig. 3 and Table 2; sample nos. 86 and 116). In addition, four tumors with ratios ≤ 2.0 exhibited increased *HER2* gene counts between ≥ 4 and < 6, corresponding to ISH group 4 (Table 2: samples 56, 78, 103, and 109). In these cases, *HER2* amplification status should have been considered questionable and been reported as negative, with a comment about the uncertainty of a response to HER2-targeted drug therapy available at the time of guidance (i.e., 2018).

**Table 2:** Focused comparison of discordantly scored BC samples and their associated FISH results

Sample	Consensus Score HercepTest (mAb)	Consensus Score pathway 4B5	IQ FISH		
			**HER2/CEP17 ratio**	**HER2 mean**	**CEP17 mean**
No. 1	2 +	1 +	1.1	2.2	2.0
No. 56	2 +	0	1.4	5.8	4.1
No. 78	2 +	1 +	1.3	4.4	3.4
No. 88	2 +	1 +	1.1	2.1	1.9
No. 97	2 +	0	1.4	3.1	2.2
No. 103	2 +	1 +	1.1	4.8	4.4
No. 104	2 +	0	1.2	3.0	2.5
No. 107	2 +	1 +	1.6	3.5	2.2
No. 108	2 +	1 +	1.6	3.6	2.3
No. 109	2 +	1 +	1.6	4.9	3.1
No. 119	2 +	1 +	1.1	3.0	2.7
No. 86	2 +	1 +	2.6	6.1	2.3
No. 116	2 +	1 +	2.4	5.4	2.3

### *HercepTest (mAb) and PATHWAY 4B5 — correlation with HER2-low status (Fig. **3B**)*

Since the development of novel HER2-directed drugs may benefit BC patients with low levels of HER2 expression (IHC 2 + /non-amplified and IHC 1 +) [10], the assay data were further analyzed with respect to their sensitivity and specificity for detecting HER2-low tumors (Fig. 3B). Out of 41 tumors (all non-amplified) that were determined to be completely negative by PATHWAY 4B5, only 19 cases (46.3%) showed no staining when using HercepTest (mAb), corresponding to the more strictly defined IHC 0 category using the Ventana score algorithm. In the remaining 22 cases, the HercepTest (mAb) stained at least some tumor cells, with approximately one-third of these cases belonging to the HER2-low group (7 × IHC 1 + , 1 × IHC 2 + non-amplified) and 14 cases in the HER2 “ultra-low” group with < 10% stained tumor cells (see also Fig. 3B, marked in grey), thus highlighting the high sensitivity of the HercepTest (mAb) used in this study.

## Discussion

Accurate assessment of HER2 status is of utmost importance for patient selection and the determination of those eligible for HER2-directed therapy. Test kits approved by the FDA have been introduced to minimize HER2 testing variability and are now recommended for use by ASCO/CAP [18]. To the best of our knowledge, this is the first study comparing the technical and diagnostic performance of the new HercepTest (mAb) with the well-established Ventana PATHWAY 4B5 test kit.

The original manual HercepTest (poly) was approved in 1998 by the FDA for assessing the eligibility of BC patients to receive trastuzumab antibody therapy. Recent reports, however, demonstrated higher specificity and sensitivity for alternative HER2 IHC assays compared to HercepTest (poly) for Autostainer [8, 11, 12]. To meet these challenges, a new HercepTest (mAb) pharmDx kit was developed that uses a licensed monoclonal antibody (clone DG44) produced with a patented process by Epitomics Inc. (an Abcam company). IHC detection of HER2 using the new HercepTest (mAb) pharmDx kit is performed on the fully automated Dako Omnis staining device. The PATHWAY 4B5 was also run on an automated staining system (i.e., the Ventana Benchmark ULTRA); however, HercepTest (mAb) performed on Dako Omnis platform, using the newly invented “dynamic gap staining technology” (reviewed in [33]), was observed to provide slightly more consistent staining as indicated by lower numbers of repetitions and fewer staining artifacts (e.g., patchy staining, edge effects, and air bubbles; see Fig. 2A, B ).

In general, IHC staining of the HercepTest (mAb) assay was characterized by distinct and sharp detection of HER2, with low to no background and/or non-specific signal detection (see also Fig. 2). Dot-like cytoplasmic staining, with or without basal membrane staining as outlined by Ventana IFU for PATHWAY 4B5, was not observed with HercepTest (mAb) in our sample series. However, a partial, mostly weak staining of normal epithelium could be seen in some samples, but was associated neither with the HER2 protein level of tumor tissue nor with false positive immunoreactions, i.e., IHC 3 + and FISH negatives as described in some cases previously for polyclonal HercepTest (poly) [29]. Instead, the comparison with FISH data demonstrated the opposite, with 100% concordance between HercepTest (mAb) and amplification status for cases scored as 0, 1 + , or 3 + by HercepTest (mAb). Two false negatives were observed using the PATHWAY 4B5 assay (1 + by PATHWAY 4B5 IHC but FISH positive), resulting in a reduced sensitivity. In this context, it should be noted that previous studies frequently reported a certain number of tumors without HER2 protein overexpression (IHC score 0 or 1 +) but being HER2 gene amplified [34–37]. While commonly considered as cases with DNA-uncoupled synthesis of HER2 protein, it might be of interest to confirm IHC score with the apparently more sensitive HercepTest (mAb).

Notably, the HercepTest (mAb) assay generated a significantly higher rate of equivocal cases (30 versus 15; see Table 1). Based on the 114 cases with available FISH data, 51.8% (14/27) were non-amplified by FISH compared to 20% (3/15) by PATHWAY 4B5. It may be argued then that HercepTest (mAb) could result in increased costs and delayed diagnosis due to an increased rate of reflex FISH testing in clinical practice. However, in light of the updated and more focused ASCO/CAP HER2 testing guideline that defines five diagnostic ISH groups [18], an in-depth analysis of FISH data revealed an additional four tumors identified by HercepTest (mAb) as IHC 2 + had increased gene counts (between ≥ 4.0 and < 6.0) and a ratio < 2.0, due to polysomy in three cases. These tumors correspond to ASCO/CAP ISH group 4 [18] and would have been classified as negative by PATHWAY 4B5 testing. The clinical implication for these patients is still not clear. The prevalence of this group in different studies varies from 1.9% [30] to 14.2% [31] and has been classified as “equivocal” in ASCO/CAP 2013 guidelines. Since 2018 [18], these cases should be reported as HER2-negative with an associated comment describing the uncertainty about patient response to HER2-directed therapies available at the time of guidance.

Meanwhile, novel HER2-directed drugs such as T-DXd have been developed using a new generation of ADCs [32]. In contrast to the first approved ADC (T-DM1) for which therapy effectiveness is still dependent on the demonstration of HER2-positive tumors (IHC3 + and/or ISH amplified), T-DXd was beneficial even after Kadcyla therapy [9]. Interestingly, there is increasing evidence that patients with HER2-low BC (HER2 2 + /non-amplified or IHC 1 + , according to ASCO/CAP 2018) also benefit from T-DXd. These new developments in HER2-targeted BC therapy have implications for both testing and the definition of HER2 sensitivity and specificity [11].

Our data demonstrate a higher detection rate of HER2 amplified breast carcinomas (100% *versus* 95%) by the HercepTest (mAb) compared to the Ventana PATHWAY 4B5 assay. In addition, the number of HER2-low expressing samples was markedly increased by using HercepTest (mAb) (35% *versus* 19%). In the upcoming era of HER2-targeted therapies administered to HER2-low BC patients [11, 34], both observations would significantly increase the number of patients eligible to HER2-directed therapies.

These promising results have already raised much interest in the scientific community focusing on the assessment of HER2-low BC in future clinical diagnostics [11]. Recent clinical trials using the HER2-directed antibody–drug conjugate T-DXd have already included patients exhibiting either IHC 1 + or 2 + /HER2 non-amplified in their HER2-low group (e.g., DB02 [NCT03523585]) or very low (“ultra-low”) HER2-expressing cohorts (HER2 IHC 0 < 1 + , weak staining in less than 10% of tumor cells, e.g., DB06 [NCT04494425]) eligible for therapy. Most recently, a large T-DXd phase III trial (DB-04) turned out to be effective in HER2-low metastatic breast cancer [38]. Thus far, these studies are based on expression analysis using the PATHWAY 4B5 antibody clone. As demonstrated in this study, the increased sensitivity of HercepTest (mAb) may allow inclusion of more patients in clinical trials, specifically by enrolling patients with HER2-low and ultra-low expression and allow the investigation of clinical response rate and outcome in these cohorts.

Another implication of testing HER2-low category of patients in this study is the accuracy of HER2 interpretation within this tumor group. Inter-rater variability was mostly restricted to the discordant assessment of HER2 0/1 + cases near the cut-off value. Future HER2 scoring will need to include more training for the HER2-low category of patients, and ASCO/CAP may refine their guidelines appropriately. Recently, French GEFPICS group published the first national recommendation for HER2 status evaluation in breast cancer with emphasis on the HER2-low concept underlining the need for harmonized testing guidelines [39]. Finally, we regard the results of this study as representative for all HER2 scores including the recently delineated HER2 low category as the carefully pre-selected case series representing the entire range of IHC scores and amplification levels, including different ISH subgroups [11, 18]. Accordingly, about 35% of cases were HER2-positive (IHC 3 + or IHC 2 + amplified) belonging to ISH group 1 (*n* = 40) and group 3 (*n* = 2). In the remaining non-amplified cases, the accuracy of assays was determined both with respect to detection of HER2-negative *versus* HER2-positive and considering HER2-low (IHC 1 + and IHC 2 + /non-amplified) and HER2-ultra-low (IHC 0 < 1 +) tumors. Therefore, our comparative study of HercepTest (mAb) with PATHWAY 4B5 addresses the main challenges that may arise during HER2 testing in BC, particularly with consideration to the emerging anti-HER2-directed drugs and patients with lower HER2 expression.

However, determining the predictive value of new HercepTest (mAb) clinical trials using this new assay is of crucial importance since more sensitive tests may not necessarily be the best predictors of response to targeted therapy. In conclusion, while both IHC assays are highly suitable for the detection of HER2 protein in BC samples, fewer assay-related failures (e.g., staining artifacts) were observed using HercepTest (mAb) Dako Omnis. The data demonstrated that HercepTest (mAb) exhibited both high specificity (100%) and high sensitivity (100%), which could be critical in patient selection for new HER2-targeting treatment options. Future studies will demonstrate whether this new assay has the capacity to provide better patient stratification, leading to better patient response rates and clinical outcomes.

## Supplementary Information

Below is the link to the electronic supplementary material.

Supplementary file1 (XLSX 19 KB)

## References

[1] Albagoush SA, Limaiem F, HER2 (2020) In: StatPearls [Internet]. Treasure Island (FL): StatPearls Publishing. Available from: https://www.ncbi.nlm.nih.gov/books/NBK537134/

[2] King CR, Kraus MH, Aaronson SA (1985). Amplification of a novel v-erbB-related gene in a human mammary carcinoma. Science.

[3] Schalper KA, Kumar S, Hui P, Rimm DL, Gershkovich P (2014). A retrospective population-based comparison of HER2 immunohistochemistry and fluorescence in situ hybridization in breast carcinomas: impact of 2007 American Society of Clinical Oncology/ College of American Pathologists Criteria. Arch Pathol Lab Med.

[4] Lal P, Salazar PA, Hudis CA, Ladanyi M, Chen B (2004). HER-2 testing in breast cancer using immunohistochemical analysis and fluorescence in situ hybridization: a single-institution experience of 2,279 cases and comparison of dual-color and single-color scoring. Am J Clin Pathol.

[5] Giuliani S, Ciniselli CM, Leonardi E, Polla E, DeCarli N, Luchini C, Cantaloni C, Gasperetti F, Cazzolli D, Berlanda G (2016). In a cohort of breast cancer screened patients the proportion of HER2 positive cases is lower than that earlier reported and pathological characteristics differ between HER2 3+ and HER2 2+/Her2 amplified cases. Virchows Arch.

[6] Ferraro E, Drago JZ, Modi S (2021). Implementing antibody-drug conjugates (ADCs) in HER2-positive breast cancer: state of the art and future directions. Breast Cancer Res.

[7] Cesca MG, Vian L, Cristóvão-Ferreira S, Pondé N, de Azambuja E (2020). HER2-positive advanced breast cancer treatment in 2020. Cancer Treat Rev.

[8] Delgado J, Vleminckx C, Sarac S, Sosa A, Bergh J, Giuliani R, Enzmann H, Pignatti F (2021) The EMA review of trastuzumab emtansine (T-DM1) for the adjuvant treatment of adult patients with HER2-positive early breast cancer. ESMO Open 6(2):100074. 10.1016/j.esmoop.2021.100074 10.1016/j.esmoop.2021.100074 PMC7920831 33647599

[9] Modi S, Saura C, Yamashita T, Park YH, Kim SB, Tamura K, Andre F, Iwata H, Ito Y, Tsurutani J, Sohn J, Denduluri N, Perrin C, Aogi K, Tokunaga E, Im SA, Lee KS, Hurvitz SA, Cortes J, Lee C, Chen S, Zhang L, Shahidi J, Yver A, Krop I (2020). DESTINY-Breast01 Investigators. Trastuzumab deruxtecan in previously treated HER2-positive breast cancer. N Engl J Med.

[10] Modi S, Park H, Murthy RK, Iwata H, Tamura K, Tsurutani J, Moreno-Aspitia A, Doi T, Sagara Y, Redfern C, Krop IE, Lee C, Fujisaki Y, Sugihara M, Zhang L, Shahidi J, Takahashi S (2020). Antitumor activity and safety of trastuzumab deruxtecan in patients with HER2-low-expressing advanced breast cancer: results from a phase Ib study. J Clin Oncol.

[11] Tarantino P, Hamilton E, Tolaney SM, Cortes J, Morganti S, Ferraro E, Marra A, Viale G, Trapani D, Cardoso F, Penault-Llorca F, Viale G, Andrè F, Curigliano G (2020). HER2-low breast cancer: pathological and clinical landscape. J Clin Oncol.

[12] Zoppoli G, Garuti A, Cirmena G (2017). Her2 assessment using quantitative reverse transcriptase polymerase chain reaction reliably identifies Her2 overexpression without amplification in breast cancer cases. J Transl Med.

[13] Press MF, Slamon DJ, Flom KJ, Park J, Zhou JY, Bernstein L (2002). Evaluation of HER-2/neu gene amplification and overexpression: comparison of frequently used assay methods in a molecularly characterized cohort of breast cancer specimens. J Clin Oncol.

[14] Miladinović M, Vučković L, Klisic A (2021) Comparison of Dako HercepTest and Ventana PATHWAY anti-HER2 (4B5) tests and their correlation with silver in situ hybridization in lung adenocarcinoma. Open Medicine 16(1):1503–1512. 10.1515/med-2021-0366 10.1515/med-2021-0366 PMC8500854 34708154

[15] Bánkfalvi A, Simon R, Brandt B, Bürger H, Vollmer I, Dockhorn-Dworniczak B, Lellé RJ, Boecker W (2000). Comparative methodological analysis of erbB-2/HER-2 gene dosage, chromosomal copy number and protein overexpression in breast carcinoma tissues for diagnostic use. Histopathology.

[16] Mayr D, Heim S, Werhan C, Zeindl-Eberhart E, Kirchner T (2009). Comprehensive immunohistochemical analysis of Her-2/neu oncoprotein overexpression in breast cancer: HercepTest (Dako) for manual testing and Her-2/neuTest 4B5 (Ventana) for Ventana BenchMark automatic staining system with correlation to results of fluorescence in situ hybridization (FISH). Virchows Arch.

[17] Lucas E, Jabbar SB, Molberg K, Fang Y, Xie XJ, Blacketer S, Sahoo S (2019). Comparison of Dako HercepTest and Ventana PATHWAY Anti-HER2 (4B5) Tests and their correlation with fluorescent in situ hybridization in breast carcinoma. Appl Immunohistochem Mol Morphol.

[18] Wolff AC, Hammond MEH, Allison KH (2018). Human epidermal growth factor receptor 2 testing in breast cancer: American Society of Clinical Oncology/College of American Pathologists Clinical Practice Guideline Focused Update. Arch Pathol Lab Med.

[19] Scott M, Vandenberghe M E, Scorer P, Boothman A-M, Barker C (2021). Prevalence of HER2 low in breast cancer subtypes using the VENTANA anti-HER2/neu (4B5) assay. J Clin Oncol.

[20] HercepTest™ mAb pharmDx (Dako Omnis). Part Number: GE001, Revision: 4, Package Inserts. Accessed 01.12.2021

[21] NordiQC (06-DEC-2021). HER2 IHC assessments in the NordiQC (Aalborg, Denmark) breast cancer module. Available: https://www.nordiqc.org/downloads/assessments/149_11.pdf. Accessed 03.01.2022

[22] Package Insert, PATHWAY anti-HER-2/NEU (4B5) rabbit monoclonal primary antibody, German, Created: 17.03.2020. Accessed 01.12.2021

[23] Jørgensen JT, Winther H, Askaa J, Andresen L, Olsen D, Mollerup J (2021). A companion diagnostic with significant clinical impact in treatment of breast and gastric cancer. Front Oncol.

[24] Rüschoff J, Hanna W, Bilous M, Hofmann M, Osamura RY, Penault-Llorca F, van de Vijver M, Viale G (2012). HER2 testing in gastric cancer: a practical approach. Mod Pathol.

[25] Scheel AH, Penault-Llorca F, Hanna W, Baretton G, Middel P, Burchhardt J, Hofmann M, Jasani B, Rüschoff J (2018). Physical basis of the ‘magnification rule’ for standardized immunohistochemical scoring of HER2 in breast and gastric cancer. Diagn Pathol.

[26] HER2 IQFISH pharmDx, Instructions for use, part number: K5731/Revision: 2/Package Inserts/Created: 12-Oct-2021. Accessed 01.12.2021

[27] Rüschoff J, Nagelmeier I, Jasani B (2021). ISH-based HER2 diagnostics. Pathologe.

[28] Nielsen SL, Nielsen S, Vyberg M (2017). Digital image analysis of HER2 immunostained gastric and gastroesophageal junction adenocarcinomas. Appl Immunohistochem Mol Morphol.

[29] Farra C, Fedda F, Tfayli A, Tawil A, Zaatari G, Ashkar H, Issa G, Boulos F (2019). The impact of partial weak staining in normal breast epithelium on the reliability of immunohistochemistry results in HercepTest-positive breast cancer. Clin Breast Cancer.

[30] Stoss OC, Scheel A, Nagelmeier I, Schildhaus HU, Henkel T, Viale G, Jasani B, Untch M, Rüschoff J (2015). Impact of updated HER2 testing guidelines in breast cancer–re-evaluation of HERA trial fluorescence in situ hybridization data. Mod Pathol.

[31] Shah MV, Wiktor AE, Meyer RG, Tenner KS, Ballman KV, Green SJ, Sukov WR, Ketterling RP, Perez EA, Jenkins RB (2016). Change in pattern of HER2 fluorescent in situ hybridization (FISH) results in breast cancers submitted for FISH testing: experience of a reference laboratory using US Food and Drug Administration Criteria and American Society of Clinical Oncology and College of American Pathologists guidelines. J Clin Oncol.

[32] Martínez-Sáez O, Prat A (2021). Current and future management of HER2-positive metastatic breast cancer. JCO Oncol Pract.

[33] Myers J (2008) A review of automated slide strainers for IHC and ISH. Med Lab Obs : 41–44. https://scholar.google.com/scholar_lookup?hl=en&publication_year=January+2008&pages=41-44&author=J+Myers&title=A+review+of+automated+slide+stainers+for+IHC+and+ISH 14758602

[34] Yamashita H, Ishida N, Hatanaka Y, Hagio K, Oshino T, Takeshita T, Kanno-Okada H, Shimizu AI, Hatanaka KC, Matsuno Y (2020). HER2 gene amplification in ER-positive HER2 immunohistochemistry 0 or 1+ breast cancer with early recurrence. Anticancer Res.

[35] Stevanovic L, Choschzick M, Moskovszky L, Varga Z (2019). Variability of predictive markers (hormone receptors, Her2, Ki67) and intrinsic subtypes of breast cancer in four consecutive years 2015–2018. J Cancer Res Clin Oncol.

[36] Dennis J, ParsaRezvaneh CT, Chau Donnie CT, Koduru P, Peng Y, Fang Y, Sarode Venetia R (2015). Quantification of human epidermal growth factor receptor 2 immunohistochemistry using the Ventana Image Analysis System. Am J Surg Pathol.

[37] Lambein K, Praet M, Forsyth R, Van den Broecke R, Braems G, Matthys B, Cocquyt V, Denys H, Pauwels P, Libbrecht L (2011). Relationship between pathological features, HER2 protein expression and HER2 and CEP17 copy number in breast cancer: biological and methodological considerations. J Clin Pathol.

[38] Modi S, Jacot W, Yamashita T, Sohn J, Vidal M, Tokunaga E, Tsurutani J, Ueno NT, Prat A, Chae YS, Lee KS, Niikura N, Park YH, Xu B, Wang X, Gil-Gil M, Li W, Pierga JY, Im SA, Moore HCF, Rugo HS, Yerushalmi R, Zagouri F, Gombos A, Kim SB, Liu Q, Luo T, Saura C, Schmid P, Sun T, Gambhire D, Yung L, Wang Y, Singh J, Vitazka P, Meinhardt G, Harbeck N, Cameron DA (2022). Trastuzumab deruxtecan in previously treated HER2-low advanced breast canceR. N Engl J Med.

[39] Franchet C, Djerroudi L, Maran-Gonzalez A, Abramovici O, Antoine M, Becette V, Berghian A, Blanc-Fournier C, Brabencova E, Charafe-Jauffret E, Chenard MP, Dauplat MM, Delrée P, Duprez-Paumier R, Fleury C, Ghnassia JP, Haudebourg J, Leroux A, MacGrogan G, Mathieu MC, Michenet P, Penault-Llorca F, Poulet B, Robin YM, Roger P, Russ E, Tixier L, Treilleux I, Valent A, Verriele V, Vincent-Salomon A, Arnould L, Lacroix-Triki M, Pour le GEFPICS. Mise à jour (2021). des recommandations du GEFPICS pour l’évaluation du statut HER2 dans les cancers infiltrants du sein en France [2021 update of the GEFPICS' recommendations for HER2 status assessment in invasive breast cancer in France]. Ann Pathol..

